# Rad54 and Rdh54 prevent Srs2-mediated disruption of Rad51 presynaptic filaments

**DOI:** 10.1073/pnas.2113871119

**Published:** 2022-01-18

**Authors:** Aviv Meir, J. Brooks Crickard, Youngho Kwon, Patrick Sung, Eric C. Greene

**Affiliations:** ^a^Department of Biochemistry and Molecular Biophysics, Columbia University, New York, NY 10032;; ^b^Department of Biochemistry and Structural Biology, University of Texas Health Science Center at San Antonio, San Antonio, TX 78229

**Keywords:** Rad51, Srs2, Rad54, Rdh54, homologous recombination

## Abstract

Srs2 is a superfamily 1 (SF1) helicase that participates in several pathways necessary for the repair of damaged DNA. Srs2 regulates formation of early homologous recombination (HR) intermediates by actively removing the recombinase Rad51 from single-stranded DNA (ssDNA). It is not known whether and how Srs2 itself is down-regulated to allow for timely HR progression. Rad54 and Rdh54 are two closely related superfamily 2 (SF2) motor proteins that promote the formation of Rad51-dependent recombination intermediates. Rad54 and Rdh54 bind tightly to Rad51-ssDNA and act downstream of Srs2, suggesting that they may affect the ability of Srs2 to dismantle Rad51 filaments. Here, we used DNA curtains to determine whether Rad54 and Rdh54 alter the ability of Srs2 to disrupt Rad51 filaments. We show that Rad54 and Rdh54 act synergistically to greatly restrict the antirecombinase activity of Srs2. Our findings suggest that Srs2 may be accorded only a limited time window to act and that Rad54 and Rdh54 fulfill a role of prorecombinogenic licensing factors.

## Significance

Homologous DNA recombination is an essential pathway necessary for the repair of double-stranded DNA breaks. Defects in this pathway are associated with hereditary breast cancer, ovarian cancer, and cancer-prone syndromes. Although essential, too much recombination is also bad and can lead to genetic mutations. Thus, cells have evolved “antirecombinase” enzymes that can actively dismantle recombination intermediates to prevent excessive recombination. However, our current understanding of how antirecombinases are themselves regulated remains very limited. Here, we study the antirecombinase Srs2 and its regulation by the recombination accessory factors Rad54 and Rdh54. Our data suggest that Rad54 and Rdh54 act synergistically to function as key regulators of Srs2, thus serving as “licensing factors” that enable timely progression of DNA repair.

Homologous recombination (HR) is a universally conserved DNA repair pathway involved in the repair of DNA double-strand breaks (DSBs) and the rescue of stalled or collapsed replication forks (1–4). The DNA repair defects associated with mutations in key HR proteins represent the cause of familial breast and ovarian cancers, as well as other diseases (5–7). HR begins with the nucleolytic resection of DSB ends to yield a long 3′ single-stranded DNA (ssDNA) overhang that becomes occupied by the heterotrimeric ssDNA-binding complex RPA (replication protein A) (8, 9). RPA is then replaced by the recombinase Rad51, which forms long helical protein filaments on the ssDNA, a key nucleoprotein intermediate known as the presynaptic complex (8, 9). Once assembled, the presynaptic complex searches for a homologous sequence and catalyzes DNA strand invasion to pair the ssDNA overhang with the homologous template to initiate repair (8, 9).

Mutations that impair HR function invariably lead to a loss of genome integrity (5–7). However, cells must balance the need to repair damaged DNA while at the same time preventing excessive HR, which otherwise has the potential to yield toxic recombination intermediates or genome rearrangements. Indeed, excessive HR can damage the genome through hyperrecombination outcomes that include illegitimate recombination, recombination between repeated elements within the genome, and the formation of inappropriate DNA structures during replication (10). To mitigate these undesirable outcomes, a number of ATP-dependent helicases down-regulate HR by physically disrupting various recombination intermediates (11, 12). Collectively, these proteins constitute a conserved group of antirecombinase enzymes that act to disrupt potentially toxic HR intermediates (11, 12). The importance of these enzymes is underscored by the prevalence of human cancers and cancer-prone syndromes associated with mutations in these enzymes (5, 13, 14).

Studies on Srs2, a superfamily 1a (SF1a) helicase in the budding yeast *Saccharomyces cerevisiae*, have furnished a general paradigm for understanding antirecombinase mechanisms and the contributions that they make to the maintenance of genome integrity (15–30). As an antirecombinase, Srs2 translocates along ssDNA while actively dismantling Rad51 protein filaments (17–19). Single-molecule studies have previously shown that Srs2 loads preferentially at clusters of RPA and/or ssDNA present at the ends of Rad51 filaments and translocates on ssDNA exclusively in a 3′ → 5′ direction (31). Multiple molecules of Srs2 can load in tandem to help remove Rad51 from the ssDNA (31). Specific protein–protein contacts between Srs2 and Rad51 help promote Rad51 removal by stimulating Rad51 ATP hydrolysis activity, leading to the formation of a Rad51–ADP complex, which has an inherently lower affinity for ssDNA compared to ATP-bound Rad51 (19). In addition to removing Rad51 from ssDNA, in vitro Srs2 can also readily remove ssDNA-bound RPA and Rad52, a well-studied recombination mediator involved in Rad51 presynaptic complex assembly, suggesting that Srs2 can act upon several ssDNA-binding proteins crucial for the early stages of HR (32, 33).

The rapid translocation velocity (∼150 nt/s) and high processivity (∼20,000 nt) of Srs2 as it dismantles Rad51 filaments in vitro raises the question of whether Srs2 itself might need to be down-regulated to allow for appropriate HR to take place (31). In fact, several regulatory mechanisms have been discovered, including direct inhibition of Srs2 translocation on ssDNA by the meiosis-specific recombinase Dmc1 (34, 35) and indirect inhibition of Srs2 activity by the recombination mediator complex Rad55–Rad57, which acts by promoting the rapid reassembly of Rad51 filaments (36). However, a potential role for Dmc1 in blocking Srs2 activity would be restricted to meiosis only (34, 35), and the effects of *rad55* or *rad57* deletions or mutations yield relatively mild phenotypes (37, 38). Rad52 has also been implicated as a potential negative regulator of Srs2 (39, 40), although we note that Srs2 can readily remove Rad52 from ssDNA in vitro (32). These observations have led us to surmise that other protein components that interact with Rad51 as part of the fully mature presynaptic complex might play a role in rendering the presynaptic complex less susceptible to disruption by Srs2.

Rad54 and Rdh54 are two closely related SNF2 superfamily DNA motor proteins that physically interact with the presynaptic complex (8, 41, 42). *S. cerevisiae* Rad54 and Rdh54 are 38% identical and 65% similar, and they are also highly conserved between yeast and humans (41, 42). Both proteins possess double-stranded DNA (dsDNA)-dependent ATPase activity and are recruited to DNA repair foci via their physical interaction with Rad51 (43). Rad54 is essential for survival in yeast exposed to DNA damaging agents (41, 42). While Rdh54 is not essential for HR, it is needed for optimal DNA repair efficiency (44–46). Rad54 and Rdh54 share a number of biochemical attributes including chromatin remodeling activity (47–50), stimulation of D-loop formation by Rad51 or Dmc1 (51–54), removal of Rad51 or Dmc1 from dsDNA (46, 55–57), and ATP-dependent motor activity on dsDNA (58–60). In addition, Rad54 acts as a motor protein that allows the Rad51 presynaptic complex to scan dsDNA for homology by a one-dimensional surveillance mechanism while promoting the separation of DNA strands in the target duplex (61), and Rdh54 appears to augment these activities by serving as an auxiliary motor (62).

Here, we have applied DNA curtains with total internal reflection fluorescence microscopy (TIRFM) to directly visualize the behavior of Srs2 on Rad51–ssDNA filaments bound by Rad54, Rdh54, or both proteins. Surprisingly, we find that either Rad54 or Rdh54 can restrict the translocation of Srs2 on ssDNA bound by Rad51. Moreover, we show that Rad54 and Rdh54 can act synergistically to block Srs2 translocation to result in potent inhibition of the antirecombinase activity of Srs2. Our findings thus support a model in which Rad54 and Rdh54 associated with the Rad51–ssDNA presynaptic complex greatly attenuate the antirecombinase activity of Srs2. We propose that the antirecombinase activity of Srs2 may be operational only within a limited time window during the earliest stages of presynaptic complex assembly. In this context, Rad54 and Rdh54 act as key licensing factors to enable the initiation of HR, and they function by restricting the antirecombinase attribute of Srs2.

## Results

### ssDNA Curtain Assay for Monitoring Rad51 Filament Stability.

**Fig. 1.**
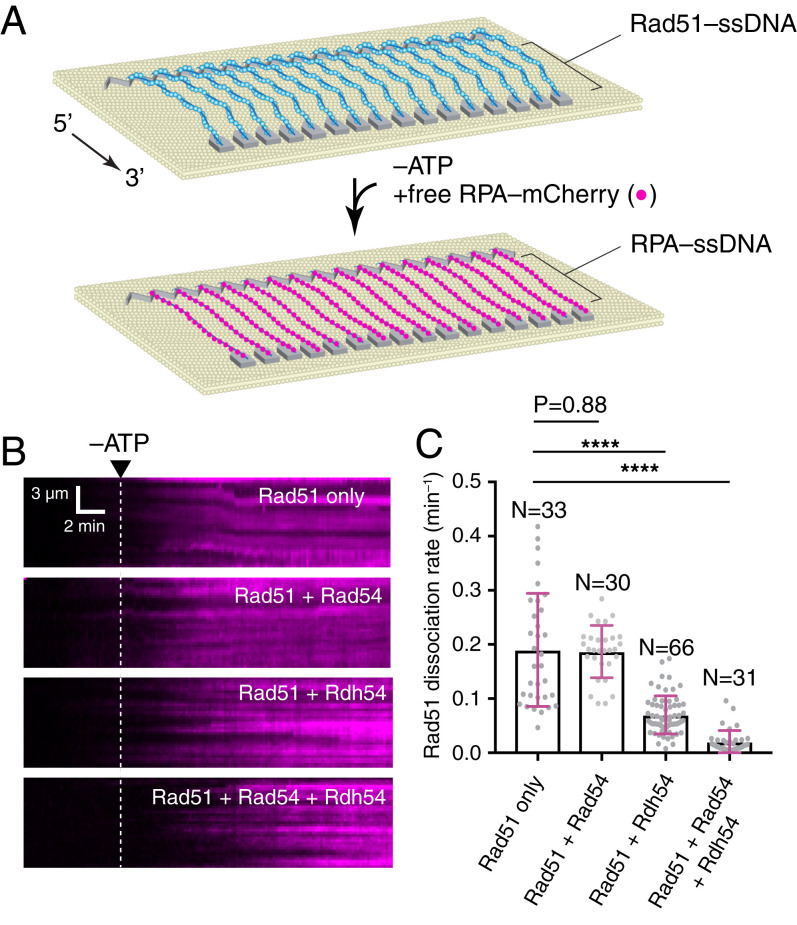
Rad54 and Rdh54 stabilize Rad51 filaments from dissociating upon depletion of ATP. (*A*) Schematic diagram of ssDNA curtain assay used to monitor Rad51 filament stability. (*B*) Representative kymographs illustrating the association of RPA–mCherry with ssDNA upon Rad51 dissociation. Reactions included Rad51 alone, Rad51 + 30 nM Rad54, Rad51 + 30 nM Rdh54, Rad51 + 15 nM Rad54, and 15 nM Rdh54, as indicated. (*C*) Measured Rad51 dissociation rates upon the depletion of ATP under each indicated reaction condition. The error bars represent the SD of the data. Asterisks above each scatter plot summarize the *P* values (unpaired *t* test); ****P < 0.0001.

We have previously established ssDNA curtain assays for visualizing the assembly and disassembly of Rad51–ssDNA filaments (Fig. 1*A*) (63–65). In these assays, Rad51 (unlabeled) is first allowed to associate with ssDNA curtains prebound with RPA–mCherry (or RPA–GFP) in the presence of 2 mM ATP (Fig. 1*A*). After assembly of the Rad51 presynaptic complex, free Rad51 is flushed from the sample chamber and replaced with buffer containing 0.5 nM free RPA–mCherry. In this setting, stable Rad51 filaments prevent the fluorescent RPA from rebinding to the ssDNA, whereas Rad51 filament dissociation is readily revealed by RPA rebinding to the ssDNA, thus providing a quantitative measure of Rad51 filament stability under any given reaction conditions (63–65). In the presence of 2 mM ATP, the Rad51 filaments remain highly stable, as we have previously reported (65), and very little rebinding of RPA–mCherry occurred. However, when ATP is removed by flushing the sample chamber with ATP-free buffer, Rad51 filaments begin to dissociate from the ssDNA, allowing RPA–mCherry to bind the ssDNA (Fig. 1 *A* and *B*). Thus, RPA–mCherry binding serves as a proxy for the dissociation of Rad51, and the rate of Rad51 filament dissociation can be determined by quantitating the on rate of RPA–mCherry, as previously described (63–65).

### Rad54 and Rdh54 Act Synergistically to Stabilize Rad51 Filaments.

We have previously established single-molecule DNA curtain assays for visualizing the binding of Rad54 and Rdh54 to both RPA–ssDNA filaments and to Rad51–ssDNA filaments (62, 66). These assays revealed that Rad54 and Rdh54 do not bind to RPA–ssDNA, which is consistent with in vivo data demonstrating that Rad54 and Rdh54 do not colocalize with repair foci until after the arrival of Rad51 (43). Moreover, Rad54 and Rdh54 can simultaneously bind to the Rad51–ssDNA filaments because they do not occupy the same binding sites within the Rad51–ssDNA filaments (62). It is not yet clear whether their presence impacts the stability of the Rad51–ssDNA filaments upon the depletion of ATP in our assays. To test the potential impact of Rad54 and Rdh54 on Rad51 filament stability, Rad51–ssDNA filaments were preassembled and then bound with Rad54, Rdh54, or both proteins. For all experiments with only Rad54 or only Rdh54, we used GFP–Rad54, GFP–Rdh54, or mCherry–Rdh54. For all experiments using both Rad54 and Rdh54, we used a combination of unlabeled Rad54 with either GFP- or mCherry-tagged Rdh54. Note that these fluorescently labeled variants have been shown to be active both in vitro and in vivo (43, 62, 66); for brevity, we will refer to the proteins as Rad54 or Rdh54.

We then compared the dissociation rate of the Rad51 filaments upon depletion of ATP for reactions containing Rad54 (30 nM), Rdh54 (30 nM), or a combination of both Rad54 and Rdh54 (15 nM each, 30 nM total). In reactions with Rad51 alone, upon ATP removal, the Rad51 filaments dissociated with a rate of 0.18 ± 0.1 s^−1^ (*n* = 33; Fig. 1 *B* and *C*). Similarly, reactions containing Rad54 yielded a Rad51 dissociation rate of 0.186 ± 0.04 s^−1^ (*n* = 30; Fig. 1 *B* and *C*). In contrast, reactions containing Rdh54 yielded a Rad51 dissociation rate of 0.063 ± 0.03 s^−1^, corresponding to a 65% reduction in the rate of Rad51 filament disassembly (*n* = 66; Fig. 1 *B* and *C*). Moreover, in reactions with both Rad54 and Rdh54, the Rad51 filaments dissociated with a rate of just 0.02 ± 0.02 s^−1^, corresponding to an 89% reduction in the rate of Rad51 filament disassembly (*n* = 31; Fig. 1 *B* and *C*). From these results, we conclude that Rad54 alone has little impact upon Rad51 filament stability upon ATP depletion, whereas Rdh54 alone affords a 2.9-fold enhancement of filament stability and Rad54 and Rdh54 act synergistically to enhance filament stability by ninefold.

### Rad54 and Rdh54 Prevent Srs2 from Inhibiting D-loop Formation.

**Fig. 2.**
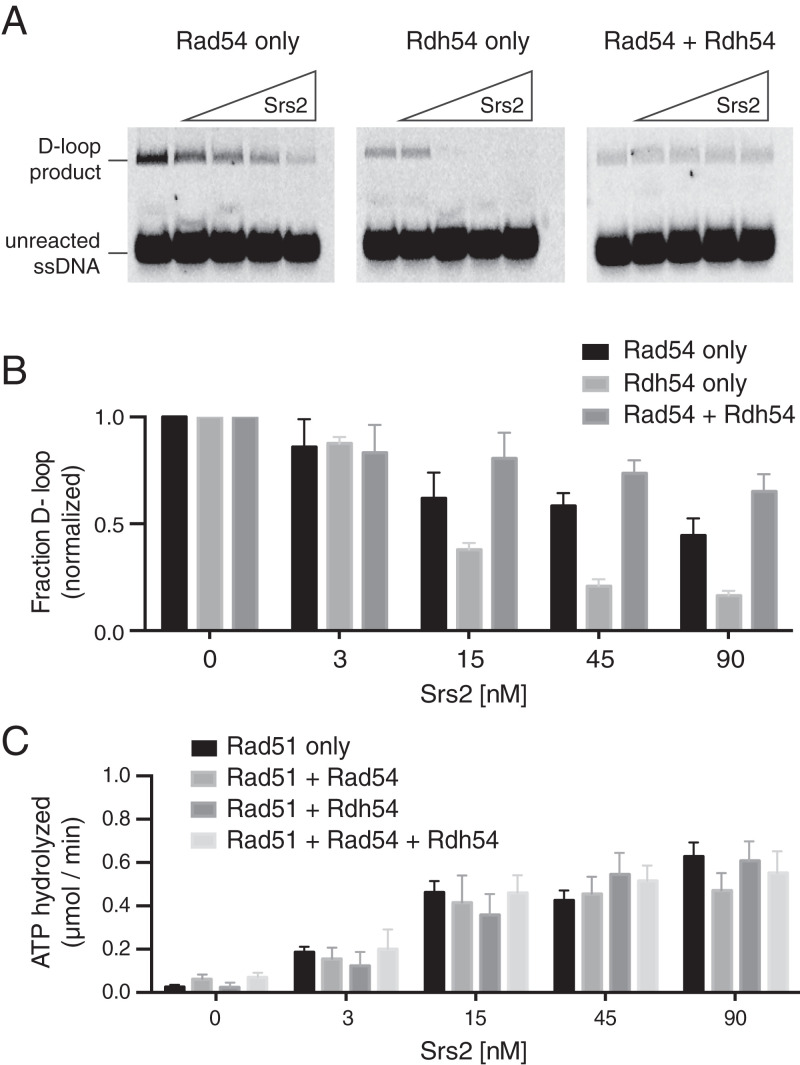
Rad54 and Rdh54 act cooperatively to allow D-loop formation in the presence of Srs2. (*A*) Assay showing Srs2-concentration–dependent inhibition of D-loop formation for reactions with 90 nM Rad54, 90 nM Rdh54, or 45 nM Rad54 + 45 nM Rdh54 (90 nM total concentration), as indicated. (*B*) Bar graph representing the fraction of D-loop formed following 10-min reactions at different concentrations of Srs2 under each given condition. The error bars represent the SD of three separate reactions. (*C*) ATP hydrolysis assay illustrating Srs2-dependent ATP hydrolysis in the presence of 30 nM Rad54, 30 nM Rdh54, or 15 nM Rad54 + 15 nM Rdh54, as indicated. The error bars represent the SD of three separate reactions.

Properly assembled Rad51 filaments are capable of pairing ssDNA with a homologous duplex target to yield a displacement loop (D-loop) (51, 53, 67, 68). Srs2 inhibits D-loop formation by virtue of its ability to remove Rad51 from ssDNA; when Srs2 is added to Rad51–ssDNA filaments before the addition of a complementary dsDNA substrate, it strips Rad51 from the ssDNA and prevents D-loop formation (17, 69). However, Srs2 does not dismantle preformed D-loops (17, 69). We therefore employed the D-loop assay to further examine the cooperative action of Rad54 and Rdh54 in attenuating the antirecombinase attribute of Srs2. For this purpose, we used an Atto-647N–labeled 90-nt ssDNA substrate that is homologous to a specific target site in the plasmid pUC19 (61, 62). Note that D-loop formation requires the simultaneous presence of Rad51 with either Rad54 or Rdh54 (51, 61, 62, 67); note also that previous studies have shown that more D-loop products are formed in reactions with Rad51 and Rad54 compared to reactions with Rad51 and Rdh54 (54). To test the effects of Rad54 and Rdh54 on Srs2-mediated inhibition of D-loop formation, the proteins were premixed with the pUC19 plasmid prior to the addition of the preassembled Rad51–ssDNA complex. For these experiments, we used 90 nM Rad54, 90 nM Rdh54, or 45 nM of each protein (i.e., 90 nM total Rad54 + Rdh54) and 0, 3, 15, 45, or 90 nM Srs2, as indicated. For all experiments reported in this study, we used a truncated version of Srs2^1–898^, which retains the same biochemical properties as full-length Srs2 but is much more soluble (19, 31); for brevity, we will refer to Srs2^1–898^ as Srs2. These assays revealed a reduction in D-loop formation under all conditions compared to reactions with no Srs2 (Fig. 2 *A* and *B*), although product formation was diminished in the presence of both Rad54 and Rdh54 compared to reactions with just Rad54. This observation was consistent with previous studies showing that the presence of Rdh54 reduces D-loop product formation in reactions with Rad54 and Rad51, albeit the molecular basis for this finding remains unknown (70). An additional contributing factor to the reduced product formation was that the concentration of Rad54 + Rdh54 was fixed at 90 nM total (i.e., 45 nM Rad54 + 45 nM Rdh54), whereas reactions with just Rad54 contained 90 nM Rad54. Reactions conducted in the absence of Srs2 yielded 31 ± 6%, 10 ± 3%, and 5 ± 2% D-loop product formation for reactions with Rad54 only, Rdh54 only, and Rad54 + Rdh54, respectively (Fig. 2*A*). Analysis of reaction products in the presence of Srs2 were normalized to account for these differences in reaction efficiencies (Fig. 2*B*). Quantification of the resulting data for reactions with Srs2 revealed a 57 ± 3% reduction in D-loop products for reactions with 90 nM Rad54 and 90 nM Srs2 and 91 ± 2% reduction in D-loop products for reactions with 90 nM Rdh54 and 90 nM Srs2, but only a 35 ± 3% reduction in D-loop products for reactions with 45 nM Rdh54, 45 nM Rad54, and 90 nM Srs2 (Fig. 2*B*). These findings suggest that Rdh54 is less adept than Rad54 at Srs2 inhibition and that Rad54 and Rdh54 act synergistically in attenuating Srs2 activity.

### Neither Rad54 nor Rdh54 Inhibit the ATP Hydrolysis Activity of Srs2.

There could be multiple mechanisms by which Rad54 and Rdh54 might attenuate the ability of Srs2 to prevent D-loop formation. For example, they might act through a mechanism similar to the meiosis-specific recombinase Dmc1, which acts as a physical barrier to Srs2 translocation on ssDNA and in doing so drastically down-regulates the ATP hydrolysis activity of Srs2 (34). If Rad54 or Rdh54 were to function similarly, then we would expect that they too would down-regulate ATP hydrolysis by Srs2. To test this possibility, we measured the effect of Rad54 and/or Rdh54 on the ATPase activity of Srs2 in assays with Rad51–ssDNA (Fig. 2*C*). Note that while Rad54 and Rdh54 both hydrolyze ATP, their activity is dsDNA-dependent. Since there was no dsDNA in these assays, we were able to selectively monitor the ATP hydrolysis activity of Srs2. Regardless, all reactions were performed in the presence and absence of Srs2 to allow us to subtract the background ATP hydrolysis activity attributable to Rad51, Rad54, and/or Rdh54 from the Srs2-containing reactions. Importantly, the presence of Rad54 or Rdh54 had no effect on the ATP hydrolysis activity of Srs2 (Fig. 2*C*), suggesting that Rad54 and Rdh54 inhibit Srs2 through a mechanism distinct from that of Dmc1.

### Rad54 Limits Srs2 Translocation on Rad51–ssDNA.

**Fig. 3.**
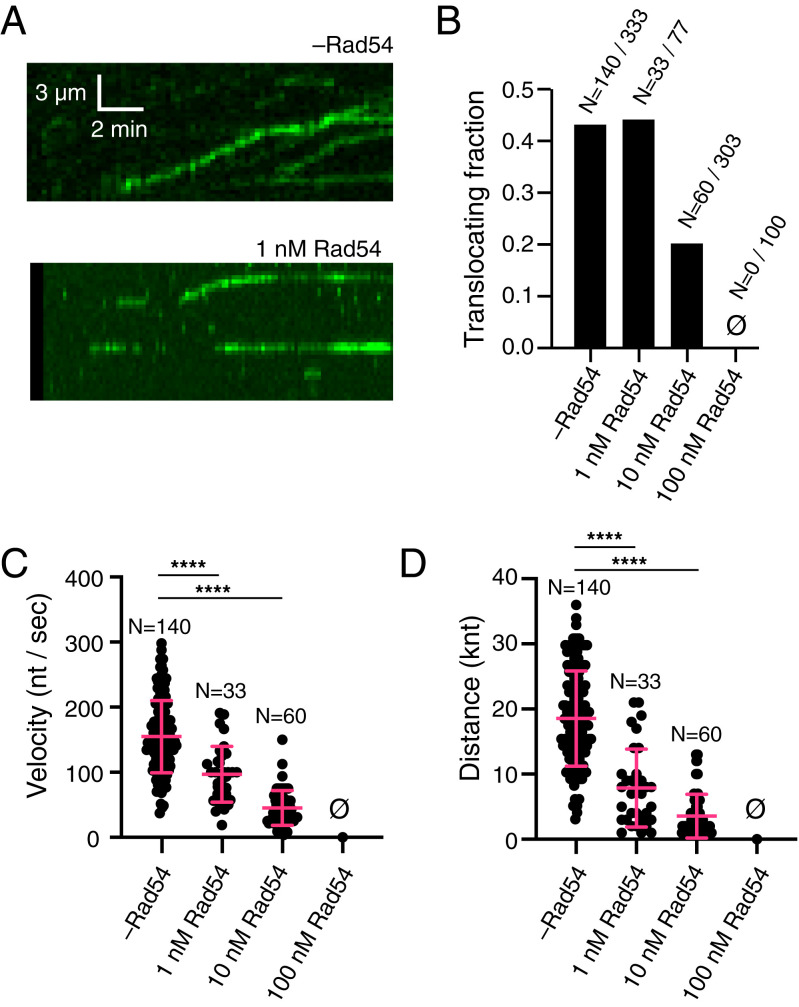
Rad54 blocks Srs2 translocation on Rad51–ssDNA. (*A*) Representative kymographs illustrating GFP–Srs2 translocation on Rad51–ssDNA filaments in the absence of Rad54 (*Top*) and in the presence of 1 nM Rad54 (*Bottom*). (*B*) Bar graph showing the fraction of active GFP–Srs2 molecules bound to Rad51–ssDNA at 0, 1, 10, or 100 nM Rad54, as indicated. (*C*) Dot plot representing the velocity of translocating GFP–Srs2 molecules at 0, 1, 10, or 100 nM Rad54. The cross bar and error bars represent the mean and SD of the data. (*D*) Dot plot showing the distance traveled by GFP–Srs2 molecules at 0, 1, 10, or 100 nM Rad54. The cross and error bars represent the mean and SD of the data. Asterisks above each scatter plot summarize the *P* values (unpaired *t* test); ****P < 0.0001. Also see *SI Appendix*, Table S1 and Fig. S1.

We have previously established ssDNA curtain assays to monitor the translocase activity of Srs2 as it disrupts Rad51 filaments bound to ssDNA (31). Here, we sought to determine whether the presence of Rad54 would influence the ability of Srs2 to disrupt Rad51 filaments. For these assays, varying concentrations of Rad54 (0, 1, 10, or 100 nM, as indicated) were preincubated with the preassembled Rad51–ssDNA filaments, and excess Rad54 was then flushed from the sample chamber, as previously described (62, 66). GFP-tagged Srs2 (0.5 nM) was then injected into the sample chamber together with 5 mM ATP and 0.5 nM unlabeled RPA, and GFP–Srs2 binding and movement on the tethered ssDNA molecules was monitored (Fig. 3*A*). We could detect GFP–Srs2 binding at all concentrations of Rad54, indicating that Rad54 does not prevent Srs2 from associating with the Rad51–ssDNA presynaptic complex. We have previously mapped the binding distribution of Srs2 on Rad51–ssDNA filaments, and we have shown that these interactions are not random (31). Instead, Srs2 preferentially loads at small clusters of RPA that remain embedded between adjacent Rad51 filaments bound to the same ssDNA molecule (31). Thus, Srs2 translocation is thought to initiate at the 3′ ends of the Rad51 filaments.

When no Rad54 was present, ∼45% of the bound GFP–Srs2 molecules underwent 3′ → 5′ translocation, consistent with our previous studies (*n* = 140/333; Fig. 3*B*) (31). A similar fraction of the bound GFP–Srs2 underwent translocation in reactions that contained 1 nM Rad54, suggesting that this low concentration of Rad54 had little impact upon the fraction of Srs2 that underwent translocation (*n* = 33/77; Fig. 3*B*). However, as the concentration of Rad54 was increased beyond 1 nM, there was a substantial decrease in the fraction of Srs2 molecules that underwent translocation. In reactions with 10 nM Rad54, only 20% of the bound GFP–Srs2 underwent translation (*n* = 60/303; Fig. 3*B*), and at 100 nM Rad54, there were no observed Srs2 translocation events (*n* = 0/100; Fig. 3*B*). From these data, we conclude that while Rad54 does not prevent Srs2 from binding to the Rad51 presynaptic complex, it can drastically reduce the number of Srs2 translocation events.

### Rad54 Can Reduce the Velocity and Processivity of Srs2.

We next determined the translocation velocity of GFP–Srs2 on ssDNA bound by either Rad51 alone or Rad51 together with varying concentrations of Rad54. In the absence of Rad54, Srs2 translocated with a velocity of 155 ± 55 nt/s, consistent with our previous findings (31). Interestingly, low concentrations of Rad54 (1 nM) had little effect upon the fraction of translocating GFP–Srs2 molecules (Fig. 3*B*); the translocation velocity itself was reduced to just 97 ± 42 nt/s, reflecting a 37% decrease in Srs2 velocity compared to reactions with no Rad54, at this low concentration of Rad54 (*n* = 33; Fig. 3*C*; *SI Appendix*, Table S1). Importantly, this inhibitory effect of Rad54 became even more pronounced with higher protein concentrations; for example, at 10 nM Rad54, the velocity of Srs2 was reduced to 45 ± 26 nt/s, corresponding to a 70% decrease (*n* = 60; Fig. 3*C*; *SI Appendix*, Table S1). As indicted above, we did not observe any Srs2 translocation in reactions with 100 nM Rad54 (Fig. 3*C*).

Rad54 also affected the distance over which Srs2 translocated on Rad51-bound ssDNA. Srs2 is highly processive in the absence of Rad54, exhibiting an average translocation distance of 18,548 ± 7,310 nt (*n* = 140; Fig. 3*D*; *SI Appendix*, Table S1), consistent with our prior studies (31). However, in reactions with either 1 nM or 10 nM Rad54, the processivity of Srs2 decreased to just 7,878 ± 5,983 nt and 3,561 ± 3,349 nt, respectively (*n* = 33 and *n* = 60; Fig. 3*D*; *SI Appendix*, Table S1). It should be noted that although we do not see measurable Srs2 translocation in assays with 100 nM prebound Rad54, it is possible that Srs2 may in fact be translocating at a very low velocity or over much shorter distances, thus eluding detection within our optical resolution limits. Nevertheless, we can conclude that Rad54 dramatically reduces the translocation velocity and processivity of Srs2 as it acts upon Rad51 filaments.

### Rdh54 Also Acts to Restrict Srs2 Translocation on Rad51–ssDNA.

**Fig. 4.**
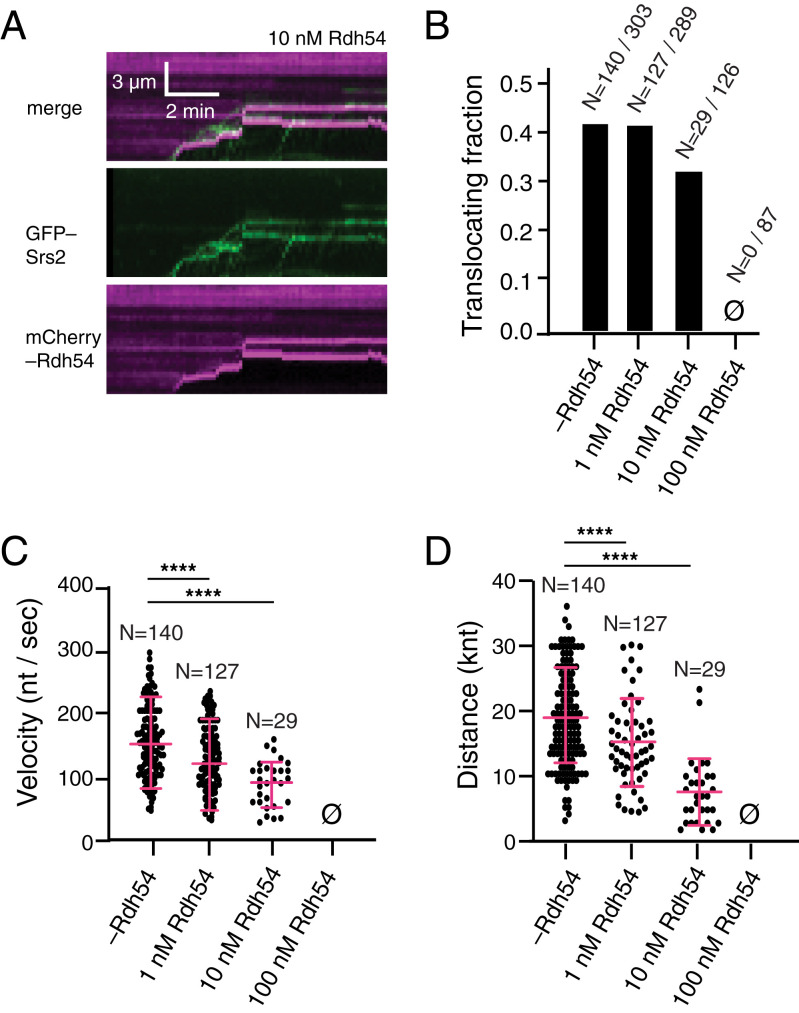
Rdh54 blocks Srs2 translocation on Rad51–ssDNA. (*A*) Representative kymographs showing GFP–Srs2 translocation on Rad51–ssDNA filaments in the presence of 10 nM Rdh54. (*B*) Bar graph comparing the fraction of translocating GFP–Srs2 on Rad51–ssDNA in the presence of varying concentrations of Rdh54, as indicated. (*C*) Velocity of individual Srs2 translocation events on Rad51–ssDNA filaments in the presence of varying concentrations of Rdh54, as indicated. The cross and error bars represent the error and SD of the data. (*D*) The distance traveled by GFP–Srs2 molecules on Rad51–ssDNA in the presence of 0, 1, 10, 100 nM Rdh54. The cross and error bars represent the mean and SD of the data. Note that the first data columns in panels *B* to *D*, corresponding to the minus Rdh54 data sets, are reproduced from Fig. 3 for comparison. Asterisks above each scatter plot summarize the *P* values (unpaired *t* test); ****P < 0.0001. Also see *SI Appendix*, Table S1 and Fig. S1.

We next asked whether Rdh54 might function similarly to Rad54 with respect to inhibition of Srs2 translocation activity on Rad51–ssDNA filaments. For these assays, varying concentrations of Rdh54–mCherry (0, 1, 10, or 100 nM, as indicated) were preincubated with the preassembled Rad51–ssDNA filaments, and excess Rdh54 was flushed from the sample chamber, as described (62, 66). GFP-tagged Srs2 (0.5 nM) was then injected into the sample chamber together with 5 mM ATP and 0.5 nM unlabeled RPA, and GFP–Srs2 binding and movement on the tethered ssDNA molecules was visualized by using TIRFM (Fig. 4*A*). We could detect GFP–Srs2 binding at all Rdh54 concentrations tested, indicating that Rdh54 does not appreciably affect association of Srs2 with the Rad51 presynaptic complex. When no Rdh54 was present in the reactions, ∼45% of the bound GFP–Srs2 molecules underwent 3′ → 5′ translocation, (*n* = 140/303; Fig. 4*B*). A similar fraction of the bound GFP–Srs2 underwent translocation in reactions that contained 1 nM Rdh54, indicating that this low concentration of Rhd54 had little or no impact upon Srs2 movement (*n* = 127/289; Fig. 4*B*). However, in reactions with 10 nM Rdh54, only 32% of the bound GFP–Srs2 underwent translocation (*n* = 29/126), and at 100 nM Rdh54, we did not observe any evidence of Srs2 translocation (*n* = 0/87; Fig. 4*B*). From these data, we conclude that even though Rdh54 does not block Srs2 binding, it does reduce the number of Srs2 translocation events. Notably, Rdh54 also exerts a striking impact upon Srs2 translocation velocity and processivity. The velocity and processivity of Srs2 was reduced to 122 ± 38 nt/s and 15,945 ± 6,621 nt in reactions with just 1 nM Rdh54 (Fig. 4*C*; *SI Appendix*, Fig. S1 and Table S1). These values decreased even further to just 79 ± 36 nt/s and 6,831 ± 5,130 nt in reactions with 10 nM Rdh54, corresponding to a 51% reduction in Srs2 velocity (*P* < 0.001) and 36% reduction in Srs2 processivity (*P* < 0.001) compared to reactions without Rdh54 (Fig. 4*C*; *SI Appendix*, Fig. S1 and Table S1). No evidence for any Srs2 translocation was observed in reactions with 100 nM Rdh54 (*n* = 0/93; Fig. 4*C*). Taken together, these data suggest that, like Rad54, Rdh54 can greatly restrict the ability of Srs2 to dismantle Rad51–ssDNA presynaptic complexes.

### Rad54 and Rdh54 Act Synergistically to Inhibit Srs2 Translocation.

**Fig. 5.**
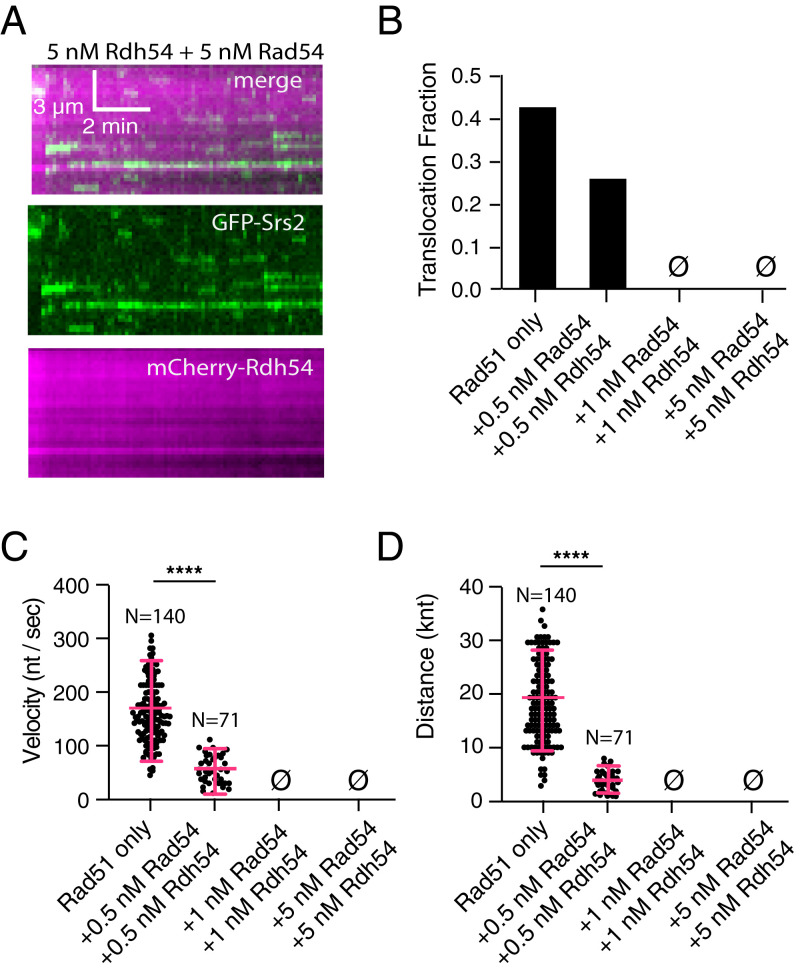
Rad54 and Rdh54 act synergistically to block Srs2 antirecombinase activity. (*A*) Representative Kymographs of GFP–Srs2 bound to Rad51–ssDNA filaments in the presence of both Rad54 and Rdh54; note that Srs2 shows no evidence of translocation under these conditions. (*B*) Bar graph comparing the fraction of translocating GFP–Srs2 on Rad51–ssDNA filaments in reactions with 5 nM Rdh54 and 5 nM Rdh54. (*C*) Dot plot representing the velocity of individual Srs2 translocation events on Rad51–ssDNA filaments in reactions with the indicated concentrations of Rad54 and Rdh54. The cross and error bars represent the error and SD of the data. (*D*) The distance traveled by GFP–Srs2 molecules in the presence of the indicated concentrations of Rad54 and Rdh54. The cross and error bars respresent the mean and SD of the data. Note that the first data columns in panels *B*–*D*, corresponding to the Rad51-only datasets, are reproduced from Fig. 3 for comparison. Asterisks above each scatter plot summarize the *P* values (unpaired *t* test); ****P < 0.0001. Also see *SI Appendix*, Table S2.

Rad54 and Rdh54 can simultaneously bind to the Rad51–ssDNA presynaptic complex (62), and, as shown above, their combined presence greatly stabilizes the presynaptic complex to dissociation upon depletion of ATP. Moreover, Rad54 and Rdh54 can each individually restrict the movement of Srs2 on Rad51–ssDNA. Therefore, we next asked whether the presence of both Rad54 and Rdh54 might provide an even greater hindrance to Srs2 translocation. For these measurements, we initially incubated 0.5 nM Rad54 plus 0.5 nM Rdh54 (for a total concentration of 1 nM) with the preassembled Rad51–ssDNA filaments prior to the injection of GFP–Srs2. Under these conditions, only 24% of the bound GFP–Srs2 molecules underwent 3′ → 5′ translocation (*n* = 71/297; Fig. 5*B*). The combination of 0.5 nM Rad54 and 0.5 nM Rdh54 also greatly reduced the velocity and processivity of GFP–Srs2, resulting in a velocity of just 49 ± 22 nt/s and processivity of 4,152 ± 2,334 nt (Fig. 5 *C* and *D*; *SI Appendix*, Table S2). Thus, in comparison with either 1 nM of Rad54 alone or Rdh54 alone, the combination of both Rad54 and Rdh54 reveals a strong synergistic effect with respect to the inhibition of Srs2 antirecombinase activity (Fig. 5 *A–C*; *SI Appendix*, Table S2).

Higher concentrations of Rad54 and Rdh54 had an even more pronounced effect on Srs2 activity. We detected no evidence for any Srs2 translocation along the Rad51–ssDNA filaments when either 1 nM or 5 nM each of Rad54 and Rdh54 were both incubated with the preassembled Rad51–ssDNA filaments (Fig. 5 *A–C*; *SI Appendix*, Table S2). Notably, even though the combined presence of either 2 nM or 10 nM total concentration of Rad54 + Rdh54 was sufficient to completely block Srs2 translocation, when assayed individually, we could detect slow Srs2 translocation at either 10 nM Rad54 or 10 nM Rdh54 (Figs. 3*D*, 4*C*, and 5*B*; *SI Appendix*, Tables S1 and S2). These findings provide further evidence that Rad54 and Rdh54 act synergistically to greatly restrict the ability of Srs2 to mediate the disruption of Rad51–ssDNA presynaptic complexes.

### Effects of Srs2 on Rad54 and Rdh54 Bound to Rad51 Presynaptic Complex.

**Fig. 6.**
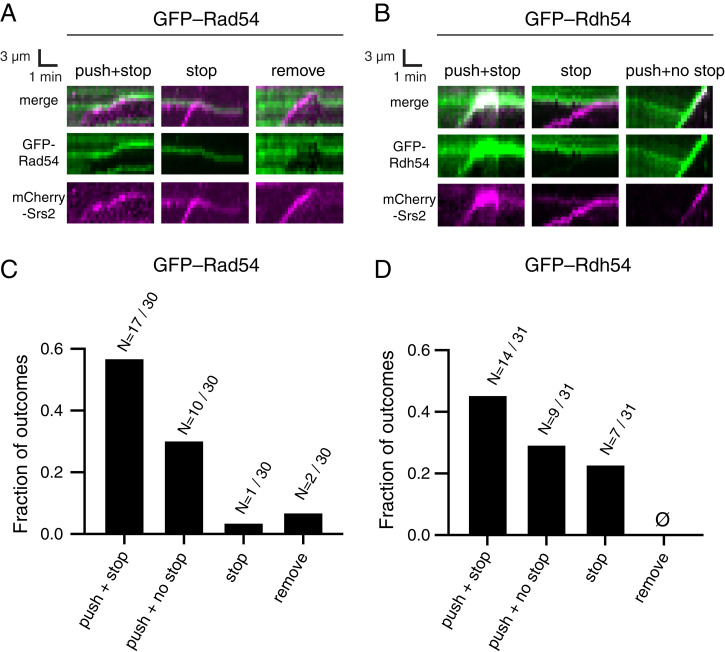
Srs2 can remodel low concentrations of Rad54- or Rdh54-bound Rad51–ssDNA filaments. (*A*) Representative kymographs illustrating the different types of outcomes for mCherry–Srs2 translocation on Rad51–ssDNA bound by 10 nM GFP–Rad54. (*B*) Representative kymographs illustrating the different types of outcomes for mCherry–Srs2 translocation on Rad51–ssDNA bound by 10 nM GFP–Rdh54. (*C*) Distribution of different outcomes for Srs2 collisions with Rad54 bound to Rad51–ssDNA filaments. (*D*) Distribution of various outcomes for Srs2 collisions with Rdh54 bound to Rad51–ssDNA filaments.

At lower concentrations of either Rad54 or Rdh54, Srs2 was able to translocate on the Rad51–ssDNA presynaptic complexes, albeit at a reduced velocity. Given these results, we sought to define how Srs2 might alter the interaction of Rad54 or Rdh54 with the Rad51 presynaptic complex. For this, we conducted two-color experiments using 0.5 nM mCherry-tagged Srs2 together with either 10 nM GFP–Rad54 or 10 nM GFP–Rdh54 bound to the Rad51–ssDNA presynaptic complexes (Fig. 6 *A* and *B*). Interestingly, we identified multiple outcomes upon collision of Srs2 with either Rad54 or Rdh54. These outcomes are 1) translocation of Srs2 is immediately halted upon encountering Rad54 or Rdh54; 2) Srs2 pushes Rad54 or Rdh54 over a short distance, then becomes halted; 3) Srs2 pushes Rad54 or Rdh54 to the end of the ssDNA without stopping; and 4) Srs2 directly removes Rad54 or Rdh54 from ssDNA (Fig. 6 *A* and *B*). Note also that a large fraction of the Srs2 molecules did not translocate, as indicated above (Figs. 3*B* and 4*B*); this immobile fraction was not analyzed further.

**Fig. 7.**
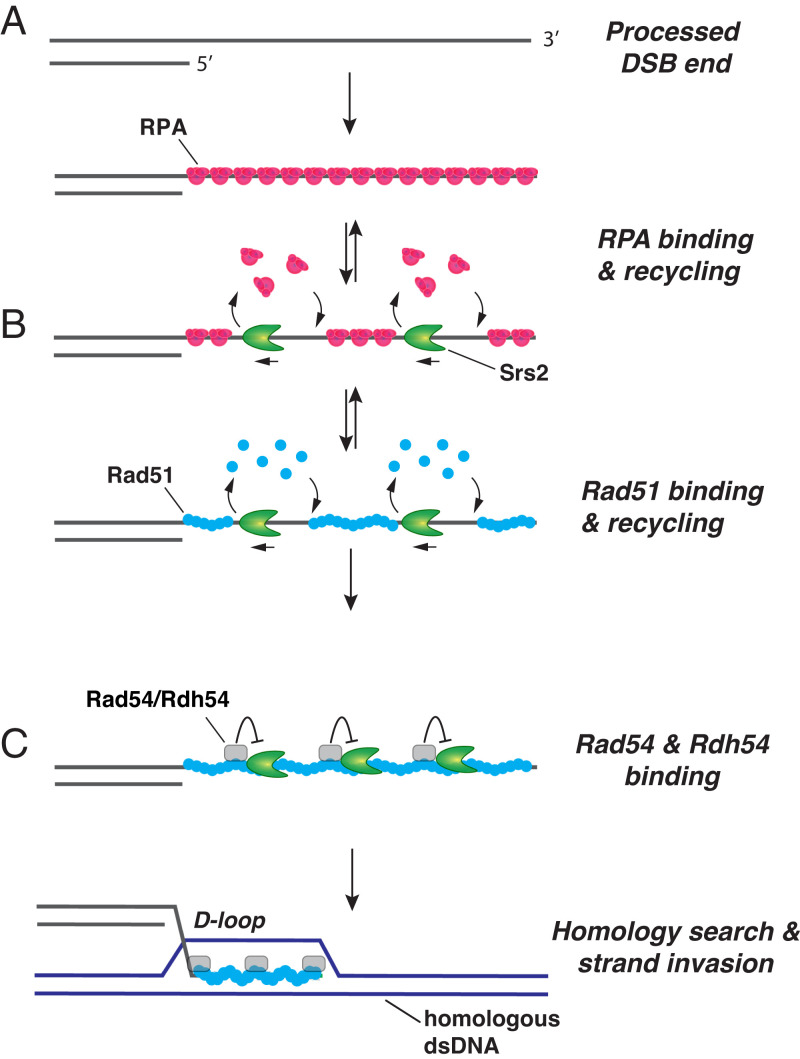
Model for protein dynamics during the early stages of homologous recombination. (*A*) DSB end processing yields long 3′ ssDNA overhangs that are quickly covered with RPA. (*B*) These earliest phases of presynaptic complex assembly are characterized by the rapid Srs2-mediated turnover of both RPA and Rad51. (*C*) Eventually, Rad54 and Rdh54 begin to accumulate on the assembling Rad51 filaments, and the presence of these two proteins marks a turning point in the reaction at which Srs2 antirecombinase activity is inhibited, allowing for subsequent steps in the reaction to take place.

In reactions with 10 nM GFP–Rad54, the most likely outcome (56% of observed collisions) was that Srs2 pushed the Rad54 along the ssDNA for a short distance but then stopped (*n* = 17/30; Fig. 6*C*). The second most common outcome (30% of observed collisions) was that Srs2 stopped translocating upon colliding with Rad54 (*n* = 10/30; Fig. 6*C*). A much smaller fraction of observed collisions either resulted in Rad54 removal from the ssDNA (6%, *n* = 2/30) or continued pushing of Rad54 without evidence of Srs2 stopping (2%, *n* = 1/30; Fig. 6*C*). In reactions with 10 nM GFP–Rdh54, the most likely outcome (44% of observed collisions) was that Srs2 pushed the Rad54 along the ssDNA for a short distance but then stopped (N= 14/31; Fig. 6*D*). The second most common outcome (29% of observed collisions) was that Srs2 stopped translocating upon colliding with Rdh54 (*n* = 9/31; Fig. 6*C*). In 22% of the observed collisions, Srs2 continued pushing Rdh54 to the end of the ssDNA without stopping (*n* = 7/31; Fig. 6*D*). Finally, we did not observe any event in which Srs2 was able to directly remove Rdh54 from the ssDNA (Fig. 6*D*). Taken together, these results suggest that when present at low concentrations, either Rad54 or Rdh54 (but not both together) presents a barrier that is partially pliable to Srs2, although the net outcome remains an overall reduction in Srs2 antirecombinase activity.

## Discussion

Here, we have investigated the relationship between the DNA motor protein Srs2, an antirecombinase enzyme that dismantles Rad51–ssDNA filaments, and the prorecombinogenic dsDNA translocases Rad54 and Rdh54, both of which bind tightly to the Rad51–ssDNA presynaptic complex. We find that Rad54 and Rdh54 act synergistically to greatly restrict the ability of Srs2 to remove Rad51 from ssDNA in vitro. From these results, we propose a model in which Rad54 and Rdh54 may act synergistically help to down-regulate the antirecombinase activity of Srs2, thus affecting the delicate balance between the pro- and antirecombination decisions in cells.

### Rad54 and Rdh54 Stabilize Rad51 Filaments upon Depletion of ATP.

Previous studies have shown that Rad54 can stabilize Rad51 filaments upon challenge with high concentrations of sodium chloride (71). Here, we use an alternative approach to assessing Rad51–ssDNA filament stability by directly monitoring Rad51 dissociation as a consequence of ATP depletion, thus mimicking the dissociation pathway that takes place upon Rad51-mediated hydrolysis of ATP to ADP plus free phosphate (see below). Interestingly, we find that Rad54 on its own does not affect the stability of Rad51 filaments; however, Rdh54 causes a 65% reduction in the Rad51 dissociation rate upon ATP depletion. Even more strikingly, the combined presence of Rdh54 and Rad54 reduces the rate of Rad51 dissociation upon ATP depletion by 89%. The DNA binding activity of Rad51 and other members of the Rad51/RecA family of DNA recombinases is tightly coupled to the ATP binding and hydrolysis cycle (72–78). Specifically, these recombinases require ATP to bind cooperatively to DNA, forming long filaments, whereas ATP hydrolysis to ADP + free phosphate (Pi) allows the proteins to dissociate from DNA, with dissociation taking place most prominently at the filament ends (72–78). Hence, HR factors that stabilize Rad51/RecA filaments may act by capping the recombinase filaments ends to prevent end-dependent monomer dissociation (77, 79). Given this general model for end-dependent Rad51 dissociation allowing for regulation through filament capping by other HR factors, our data suggest the possibility that Rad54 and Rdh54 may also help prevent Rad51 filament disassembly by capping the ends of the Rad51 filaments. Importantly, we have previously shown that Rad54 and Rdh54 can simultaneously bind to the Rad51–ssDNA filaments, and they appear to occupy distinct positions within the filaments and do not compete with one another for binding sites (62). Thus, one attractive model is that Rad54 and Rhd54 act synergistically to suppress Rad51 dissociation by capping both the 5′ and 3′ ends of the filaments, perhaps with Rad54 and Rdh54 acting at opposite ends of the filaments. However, we caution that this model is speculative, and high-resolution structural data would be needed for precisely determining the relative locations of Rad54 and Rdh54 within the Rad51–ssDNA filaments.

### Rad54 and Rdh54 Block the Antirecombinase Activity of Srs2.

Our data reveal that Rdh54 and Rad54 act synergistically in preventing Srs2-mediated disruption of Rad51 filaments. Notably, Srs2 translocation becomes completely suppressed in reactions in which just 1 nM of Rad54 and 1 nM Rdh54 (2 nM total) were preincubated with the Rad51 filaments, as compared to the finding that 10 nM of either Rad54 or Rdh54 alone slows but does not fully inhibit Srs2 antirecombinase activity. We have previously shown that a Rad54 or Rdh54 concentration of either 5 nM or 10 nM is insufficient for fully saturating the Rad51–ssDNA filaments (62, 66). These findings thus suggest a striking level of synergy between Rad54 and Rdh54 with respect to Srs2 inhibition. When tested individually, at a concentration of 100 nM protein, both Rad54 and Rdh54 could completely block Srs2 translocation; note that at this concentration, the Rad51–ssDNA filament should be fully saturated with Rad54 or Rdh54 (62, 66). Interestingly, at lower concentrations, Rad54 was somewhat more effective at inhibiting Srs2 translocation relative to Rdh54 (Figs. 3 *C* and *D* and 4 *C* and *D*; *SI Appendix*, Fig. S1 and Table S1). One possible explanation for this differential effect is that, when assayed at the same protein concentration, there may be more Rad54 bound to the Rad51–ssDNA filaments than Rdh54, or Rad54 may simply associate with the Rad51 filaments more stably. We disfavor both of these hypotheses because our previous studies have shown that Rad54 and Rdh54 exhibit very similar Rad51–ssDNA binding characteristics (62, 66). An alternative explanation may be that the unique spatial distributions of Rad54 and Rdh54 within the Rad51–ssDNA filaments could influence their relative effectiveness at blocking Srs2 translocation. For instance, Srs2 translocates in the 3′ to 5′ direction, so if Rad54 localizes preferentially to the 3′ end of the Rad51 filaments, it could impart an advantage in preventing the accumulation of Srs2 molecules. Indeed, the possibility that Rad54 and Rdh54 together can cap both the 3′ and 5′ ends of the Rad51 filaments may help to explain their synergistic effects on Rad51 filament stability upon ATP depletion and restriction of Srs2 antirecombinase activity.

### Distinct Strategies for Srs2 Down-Regulation.

Based on our previously published work and results presented here, we propose that there exist multiple strategies for restricting the antirecombinase attribute of Srs2. We have previously shown that the meiosis-specific recombinase Dmc1 is also able to counteract the antirecombinase activity of Srs2 (34) in concordance with in vivo observations (35). In this case, ssDNA-bound Dmc1 filaments completely block Srs2 translocation and strongly inhibit Srs2-mediated ATP hydrolysis in vitro (34). This mechanism contrasts with the regulatory effects we document here for Rad54 and Rdh54, which block Srs2 translocation but appear to have little or no effect on its ATPase activity, suggesting the possibility that Srs2 remains bound to ssDNA but undergoes futile cycles of ATP hydrolysis that are decoupled from protein translocation. The antirecombinase activity of Srs2 can also be counteracted by the Rad51 paralog complex Rad55–Rad57 (37, 38). In this case, our studies suggest that Rad55–Rad57 does not present a physical blockage to Srs2 movement, but instead acts by promoting the rapid reassembly of Rad51 filaments after their initial disruption by Srs2 (36). Thus, Rad54 and Rdh54 act synergistically to counteract Srs2 antirecombinase activity through a unique molecular mechanism that is distinct from both Dmc1 and Rad55–Rad57. It is possible that similar sets of regulatory strategies exist for other ATP-dependent antirecombinases, such as human FBH1 and RECQ5.

### Protein Dynamics During the Early Stages of Presynaptic Complex Assembly.

In HR, the ends of DSBs are processed to yield long 3′ ssDNA overhangs that serve as a platform for the ordered recruitment of proteins necessary for assembly of the presynaptic complex (Fig. 7). The assembly process begins with the binding of RPA (Fig. 7*A*), followed closely by RPA-interacting factors such as Rad52 and Mec1–Ddc2 (1–4). Next comes Rad51, together with the Rad51 paralog complex Rad55–Rad57, which helps to promote Rad51 assembly on the RPA-bound ssDNA (Fig. 7*B*) (1–4). Assembly of the Rad51 filaments allows for the subsequent recruitment of Rad51-binding proteins such as Rad54 and Rdh54 (Fig. 7*C*) (1–4). The antirecombinase Srs2 is also involved during these early presynaptic complex assembly steps (1–4), a premise that is consistent with the presence of Srs2 in DNA damage-induced foci even when Rad51 is absent (80). Studies of Srs2 indicate that it readily removes RPA, Rad52, and Rad51 from ssDNA (17–20, 31–33). Rad55–Rad57 helps to offset Rad51 removal by enhancing Rad51 rebinding, but it does not block Srs2 activity (36). These findings thus reveal an important role of Srs2 in controlling the stability of early HR intermediates, and they also raise a question concerning how the HR machinery manages to overcome the restrictive action of Srs2 in order to proceed to subsequent steps in the break–repair process. Importantly, our data suggest that Rad54 and Rdh54 fulfill a HR licensing role by blocking the ability of Srs2 to translocate on the Rad51-containing presynaptic complex, thus allowing for timely D-loop formation. In this regard, it should be noted that Rad54 and likely Rdh54 also serve to promote DNA homology search and DNA strand invasion (51–54, 61, 62). Thus, the binding of Rad54 and Rdh54 to the Rad51–ssDNA filament may represent a point of no return that commits repair to the HR pathway.

Our results also raise the question of why Srs2 drives general protein turnover from ssDNA during early HR stages. We surmise that this remarkable attribute of Srs2 helps to eliminate potentially toxic nucleoprotein intermediates that would otherwise be fed into the HR pathway. For instance, timely Srs2 action would help dismantle Rad51–ssDNA filaments to prevent Rad54 and Rdh54 accumulation at inappropriate locales such as replication forks or ssDNA gaps (16, 21, 26–30). We note that Rad54 overexpression is toxic, and this deleterious effect is further exacerbated in cells treated with the DNA alkylating agent methyl-methanesulfonate (81, 82). These findings suggest that endogenous levels of Srs2 may be insufficient to prevent excessive recombination under these circumstances.

Our study unveils a mechanism of HR regulation by Rad54 and Rdh54. It will be important to determine whether an analogous regulatory mechanism is also operational in human cells and its relevance to the maintenance of genome integrity.

## Methods

### Protein Purification.

Rad51, RPA, Srs2, Rad54, and Rdh54 were purified as previously described (31, 32, 61).

### D-loop Assays.

D-loop experiments were performed in HR buffer (30 mM Tris-OAc [pH 7.5], 50 mM KCl, 20 mM MgOAc, 1 mM dithiothreitol [DTT], 0.2 mg/mL bovine serum albumin [BSA]) using a 90-nt Atto-647N ssDNA substrate that was homologous for a region on the pUC19 plasmid, as previously described (61, 67). Rad51 (300 nM) was incubated with the 90-mer substrate (10 nM) in HR buffer + 2 mM ATP at 30 °C for 15 min. The preformed Rad51 filaments were then mixed with 30 nM Rad54 or Rdh54 (as indicated), 500 nM RPA, and pUC19 plasmid (3 nM plasmid). Following a 10-min incubation, reactions were quenched with the addition of an equal volume of Stop buffer (20 mM ethylenediaminetetraacetic acid [EDTA], 1% sodium dodecyl sulfate [SDS], and 20% glycerol). Reactions were deproteinized by the addition of proteinase K (0.5 mg/mL, final concentration), and the DNA products were resolved on a 0.9% agarose gel in 1×Tris acetate-EDTA (TAE) buffer. Gels were scanned using a Typhoon imager with a 635-nm laser (GE Health Sciences).

### ATP Hydrolysis Assays.

ATP hydrolysis assays were performed using a malachite green‐based kit (Sigma, Cat. No. MAK113) in accordance with the manufacturer’s instructions. Reactions were initiated by the addition of ATP (500 nM) to a reaction mix containing 100 ng ssDNA (ΦX174 RF II; 5,386 bp; New England Biolabs, catalog no. N3022L), 50 nM RPA, and 50 nM Rad51 with total Rad54 only, Rdh54 only, or Rad54 + Rdh54 concentration of 30 nM. Reactions were terminated at the indicated time points by addition of 100 μL of the malachite green reagent solution to 5 μL of the reaction sample in a 96‐well microplate (Greiner Bio One, catalog no. 655096). After a 20-min incubation at room temperature, the absorbance at 620 nm was measured on a Spectra Max M5e multimode reader (Molecular Devices). Standard solution was used to calculate the amount of free phosphate present in each sample.

### Single-Molecule Imaging.

All experiments were conducted with a custom-built prism-type total TIRF microscope (Nikon) equipped with a 488-nm laser (Coherent Sapphire, 200 mW), a 561-nm laser (Coherent Sapphire, 200 mW), and two Andor iXon electron-multiplying charge-coupled device (EMCCD) cameras (63, 83).

### Flow Cell Construction.

Chrome barriers were deposited on quartz microscope slides via e-beam lithography and thermal evaporation, as described (63, 84). Lipid bilayers were prepared with 91.5% 1,2-dioleoyl-*sn*-glycero-3-phosphocholine, 0.5% 1,2-dioleoyl-*sn*-glycero-3-phosphoethanolamine-*N*-(cap biotinyl), and 8% 1,2-dioleoyl-*sn*-glycero-3-phosphoethanoloamine-*N*-[methoxy(polyethyleneglycol)-2,000] (Avanti Polar Lipids, Inc., catalog nos. 850375P, 870273P, and 880130P, respectively). Lipid bilayers were deposited in preformed flow chambers through sequential deposition of a lipid master mix in lipid buffer (20 mM Tris-Cl [pH 7.5], 100 mM NaCl).

### Data Acquisition and Analysis.

All data were collected with a 100-ms integration time, and laser shuttering was varied to minimizing photobleaching. Images were collected using Nikon software, and images were exported as individual TIFF images as described (63, 83). TIFF stacks were imported into ImageJ (Fiji). For two-color imaging, the two channels were first corrected for stage drift and then merged into TIFF images, which were then converted to TIFF stacks. All TIFF stacks were then corrected for stage drift using the registration/translation function within Fiji (83). For each time course experiment, kymographs were generated from the TIFF image stacks by defining a 1-pixel–wide region of interest (ROI) along the axis of each individual ssDNA molecule, and these ROIs were extracted from each image within the TIFF stack (83). All of the slices corresponding to one ssDNA molecule were then aligned to yield a kymograph representing the entire experimental time course, and this process was repeated for each ssDNA molecule that was analyzed (83).

### ssDNA Curtains and Rad51 Filament.

ssDNA was generated by rolling circle replication using phi29 DNA polymerase with a biotinlyated primer annealed to M13 circular ssDNA as a template (63, 83). The ssDNA was tethered to the bilayer through a biotin–streptavidin linkage, as described (63, 83). The ssDNA molecules were aligned at a flow rate of 0.5 mL/min in HR buffer + RPA (30 mM Tris-OAc [pH 7.5], 50 mM KCl, 20 mM MgOAc_2_, 0.2 mg/mL BSA, 1 mM DTT, 0.1 nM RPA). Once ssDNA molecules were aligned, the flow rate was adjusted to 1.0 mL/min, and 0.5 mL of 7 M urea was injected into the flow cell to further extend the ssDNA. HR buffer + RPA was then flushed through the sample chamber at 1.0 mL/min for an additional 10 min. The sample chamber was then flushed with HR buffer + ATP (30 mM Tris-OAc [pH 7.5], 50 mM KCl, 20 mM MgOAc_2_, 0.2 mg/mL BSA, 1 mM DTT, 2.5 mM ATP) at 1.0 mL/min for 3 min. Rad51 (1 μM) was injected into the flow cell, flow was stopped, and the reactions were incubated at 30 °C for 15 min. When GFP- or mCherry-tagged RPA was used, the Rad51 filament assembly was monitored with the appropriate laser at an image acquisition rate of 3 frames/min. After 15 min, free Rad51 was removed with HR buffer + ATP. Protein binding experiments with Rad54, Rdh54, and Srs2 were conducted in HR buffer plus ATP.

### Rad51 Filament Disassembly Experiments.

Rad51–ssDNA filaments were preassembled in HR buffer in the absence of RPA as described above. RPA–mCherry (100 pM) was then injected in HR buffer + 5 mM ATP at 0.2 mL/min for 10 min. The experiment was monitored at a frame rate of 1 frame per 20 s. After 10 min, flow was switched to HR buffer plus RPA–mCherry − ATP, and the binding of RPA–mCherry to the ssDNA, which represents the dissociation of Rad51, was monitored for an additional 45 min. For data analysis, kymographs were generated as described above. The increase in RPA–mCherry intensity was measured for each kymograph and normalized by setting the highest value in each kymograph to 1 and normalizing all other signals to that value, generating an association curve. The association rate was then determined by fitting the normalized association data for each molecule using the single-phase association curve equation in GraphPad Prism, as described (62, 66). The mean and SD of the data were then calculated from the pooled values.

### Srs2 Translocation Experiments.

For single-molecule Srs2 translocation experiments, Rad51–ssDNA complexes were preformed, and then Rad54, Rdh54, or both were allowed to bind for 10 min in HR buffer − RPA. After 10 min, excess Rad54, Rdh54, or both were removed by flushing the sample chamber with HR buffer + 5 mM ATP at 1 mL/min for 2 min. GFP- or RFP-tagged Srs2 (1 to 898; 500 pM; Refs. 31, 32) was then injected into the sample chamber in HR buffer + 5 mM ATP + 500 pM RPA. Flow was terminated, and the reaction was observed for 10 to 20 min with at a frame rate of 1 frame per 10 s. Srs2 translocation events were analyzed as previously described (31, 32, 83). Briefly, velocities and distances traveled were calculated from the kymographs and values calculated as follows: $\mathrm{velocity}=\;\left[\left(Y_{f}-\;Y_{i}\right)\times 1000\;nt\right]/\left[\left(X_{f}-X_{i}\right)\times \mathrm{frame}\;\mathrm{rate}\right]$ and $\mathrm{distance}=\;\left(Y_{f}-Y_{i}\right)\times 1000\;nt$, where $Y_{i}$ and $Y_{f}$ correspond to the initial and final positions of Srs2 along the ssDNA and $X_{i}$ and $X_{f}$ correspond to the initial and final frame number. The mean velocities and distance traveled were then calculated from the distribution of the data.

## Supplementary Material

Supplementary File

## Data Availability

All study data are included in the article and/or *SI Appendix*.
