# Supplementary material for: Rad54 and Rdh54 prevent Srs2-mediated disruption of Rad51 presynaptic filaments
Source: Proc Natl Acad Sci U S A. 2022 Jan 18;119(4):e2113871119. doi: 10.1073/pnas.2113871119 (PMC8795549; doi:10.1073/pnas.2113871119)
Supplement: Supplementary File [file pnas.2113871119.sapp.pdf]

Supplementary Figure S1.

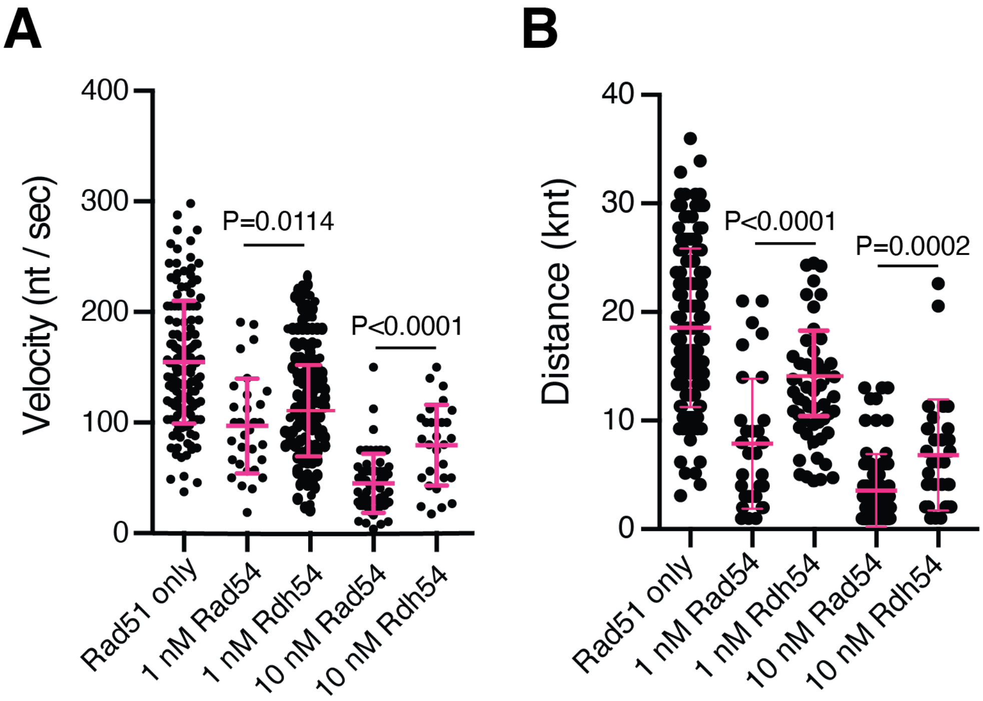

Figure S1. Side-by-side comparison of Srs2 translocation velocity and processivity in reactions with Rad54 or Rdh54. (A) The data shown here reproduce the Srs2 translocation data already shown in Figures 3C and 4C and **(B)** the Srs2 processivity data shown in Figures 3D and 4D. The P values (unpaired *t* test) indicate pairwise comparisons of the Srs2 translocation data for each indicated concentration of Rad54 and Rdh54.

Supplementary Table S1. Velocity and processivity statistics for reactions with only Rad54 or only Rdh54.

**Fig. 3C**	**mean**	**std dev**	**N**	**P value**
–Rad54	155 nt/sec	± 55 nt/sec	140	N.A.
1 nM Rad54	97 nt/sec	± 42 nt/sec	33	<0.0001
10 nM Rad54	45 nt/sec	± 26 nt/sec	60	<0.0001
100 nM Rad54	∅	N.A.	N.A.	N.A.

**Fig. 3D**	**mean**	**std dev**	**N**	**P value**
–Rad54	18.6 knt	± 7.3 knt	140	N.A.
1 nM Rad54	7.9 knt	± 6.0 knt	33	<0.0001
10 nM Rad54	3.6 knt	± 3.4 knt	60	<0.0001
100 nM Rad54	∅	N.A.	N.A.	N.A.

**Fig. 4C**	**mean**	**std dev**	**N**	**P value**
–Rdh54	155 nt/sec	± 55 nt/sec	140	N.A.
1 nM Rdh54	122 nt/sec	± 38 nt/sec	127	<0.001
10 nM Rdh54	79 nt/sec	± 36 nt/sec	29	<0.0001
100 nM Rdh54	∅	N.A.	N.A.	N.A.

**Fig. 4D**	**mean**	**std dev**	**N**	**P value**
–Rdh54	18.6 knt	± 7.3 knt	140	N.A.
1 nM Rdh54	15.9 knt	± 6.6 knt	127	<0.001
10 nM Rdh54	6.8 knt	± 5.1 knt	29	<0.0001
100 nM Rdh54	∅	N.A.	N.A.	N.A.

Supplementary Table S2. Velocity and processivity statistics for reactions with mixtures of Rad54 and Rdh54.

Fig. 5C	mean	std dev	N	P value
–Rad54, –Rdh54	155 nt/sec	± 55 nt/sec	140	N.A.
0.5 nM Rad54, 0.5 nM Rdh54	49 nt/sec	± 22 nt/sec	71	<0.0001
1.0 nM Rad54, 1.0 nM Rdh54	∅	N.A.	N.A.	N.A.
2.5 nM Rad54, 2.5 nM Rdh54	∅	N.A.	N.A.	N.A.
5.0 nM Rad54, 5.0 nM Rdh54	∅	N.A.	N.A.	N.A.

Fig. 5D	mean	std dev	N	P value
–Rad54, –Rdh54	18.6 knt	± 7.3 knt	140	N.A.
0.5 nM Rad54, 0.5 nM Rdh54	4.2 knt	± 2.3 knt	71	<0.0001
1.0 nM Rad54, 1.0 nM Rdh54	∅	N.A.	N.A.	N.A.
2.5 nM Rad54, 2.5 nM Rdh54	∅	N.A.	N.A.	N.A.
5.0 nM Rad54, 5.0 nM Rdh54	∅	N.A.	N.A.	N.A.
